# Factors associated with adherence to gluten‐free diet in patients with concomitant celiac disease and type 1 diabetes

**DOI:** 10.1002/jpr3.70231

**Published:** 2026-08-14

**Authors:** Laura Kallio, Sofia Kröger, Riku Tauschi, Oona Kähkönen, Linnea Aitokari, Katri Kaukinen, Laura Kivelä, Kalle Kurppa, Anna Eurén

**Affiliations:** ^1^ Celiac Disease Research Center Tampere University and Tampere University Hospital Tampere Finland; ^2^ Tampere Center for Child, Adolescent, and Maternal Health Research Centre for Child Health Research Tampere University Tampere Finland; ^3^ Valkeakoski Social and Health Care Centre Wellbeing Services County of Pirkanmaa Valkeakoski Finland; ^4^ Department of Internal Medicine Tampere University Hospital Tampere Finland; ^5^ Children's Hospital, and Pediatric Research Center University of Helsinki and Helsinki University Hospital Helsinki Finland; ^6^ Children's Hospital at Tampere University Hospital Tampere Finland; ^7^ Department of Pediatrics, Seinäjoki Central Hospital The University Consortium of Seinäjoki Seinäjoki Finland

**Keywords:** compliance, nutrition, screening, treatment

## Abstract

Celiac disease (CeD) and type 1 diabetes (T1D) often coexist. The burden of concomitant conditions may predispose CeD patients to impaired adherence to a gluten‐free diet (GFD), but the factors predisposing to dietary lapses remain unclear. We compared the baseline characteristics and follow‐up data between adherent and nonadherent CeD children with a dual diagnosis (*n* = 69). Overall, 72% were found to be adherent, while 28% were nonadherent. Adherent patients were detected significantly more often in annual CeD screening than by symptoms, whereas the groups did not differ in sex, familial risk for CeD or T1D, presence of other diseases, age at T1D and CeD diagnoses, or time between diagnoses, severity of clinical symptoms, or duodenal lesion at CeD diagnosis, or CeD autoantibody levels at diagnosis or during treatment. Dietary adherence is suboptimal in CeD patients with concomitant T1D, but a CeD diagnosis via systematic screening does not predispose to this.

## Abstract (highlights)

**What is Known:**

- Celiac disease (CeD) and type 1 diabetes (T1D) often co‐occur due to shared genetic risk factors.
- Multiple autoimmune conditions may predispose to a greater disease burden and impair adherence to gluten‐free diet (GFD).

**What is New:**

- Almost 30% of children with CeD and T1D were nonadherent.
- Adherent patients were detected more often in annual CeD screening, whereas a greater proportion of nonadherent patients were found based on symptoms.
- The clinical presentation of CeD is comparable between patients with strict dietary adherence and those with lapses, both at diagnosis and during follow‐up.

## 1 INTRODUCTION

Celiac disease (CeD) and type 1 diabetes (T1D) often co‐occur due to shared risk factors. The main external driver in CeD, dietary gluten, has been identified, enabling treatment with a gluten‐free diet (GFD).^1^ In contrast, patients with T1D require lifelong insulin replacement therapy.^2^ Specific serum autoantibodies are produced in both conditions, but tissue transglutaminase antibodies (TGA) in CeD enable noninvasive case‐finding and, despite suboptimal sensitivity, may also help assess adherence to GFD. Due to its diverse clinical presentation, CeD remains heavily underrecognized, which has led to the common practice of systematically testing T1D patients for CeD.^1^

The benefits of active screening are not straightforward, as both conditions are challenging to treat and cope with. Although the treatment of T1D is evolving rapidly, the disease still requires continuous care, including carbohydrate counting and insulin dosing.^2^ Similarly, a strict GFD in CeD can be difficult to maintain and socially restrictive, all of which predispose to dietary lapses. In this regard, CeD patients with coexisting T1D, often detected through screening, could form a special risk group.^3^, ^4^ Ascertaining whether the diagnostic strategy or other factors, such as age and clinical presentation at diagnosis,^3^, ^4^, ^5^, ^6^ affect the success of GFD could help improve treatment outcomes^7^ and estimate the pros and cons of screening programs.

Both CeD and T1D are common in Finland^1^ and regular screening for CeD in children with T1D has long been carried out. These aspects motivated us to study overall adherence to GFD, as well as the role of screening‐detected diagnosis and other factors potentially associated with nonadherence in children with coexisting T1D and CeD.

## 2 METHODS

### 2.1 Ethics statement

Permission to collect the patient record data was obtained from the Tampere University Hospital. No ethics committee statement was required according to the national regulations, since this was a register‐based study and none of the patients were contacted. All analyses were conducted without personal identifying information.

### 2.2 Study design and patients

The study was conducted at Tampere University between June 1st, 2020 and April 2025. Medical information was collected from systematically maintained patient records. The patients were identified through data extraction using international classification of diseases 9th revision (ICD‐9) (250.00) and internal classification of diseases 10th revision (ICD‐10) (K90.0, E10.9) codes and from the catchment area of one center covering approximately 1 million residents. Inclusion criteria were a confirmed diagnosis of T1D and CeD, availability of appropriate medical data at diagnosis, and sufficient follow‐up data on CeD. Patients with a CeD diagnosis occurring before the T1D diagnosis were excluded due to insufficient follow‐up data (Supporting Information S1: Figure S1). The patients were further categorized into two subgroups based on adherence or nonadherence to GFD.

### 2.3 Clinical data

Demographic data, age at CeD and T1D diagnosis, family history of CeD and T1D, presence of other comorbidities, and clinical presentation of CeD at diagnosis were collected. Clinical presentation of CeD was further classified into gastrointestinal (e.g., diarrhea, abdominal pain, constipation, bloating), extraintestinal (e.g., growth failure, arthralgia, anemia), and screen‐detected (annual TGA screening due to previously diagnosed T1D). Severity of symptoms was categorized into severe, moderate, mild, and asymptomatic.^8^

### 2.4 CeD serology and histology

The results for CeD serology and duodenal histology were collected at diagnosis and during follow‐up on GFD as available. Serum immunoglobulin A (IgA) class endomysial antibodies (EmA) were measured by an indirect immunofluorescence method. Dilution of 1: > 5 was considered positive and was further diluted until 1:4000 or negative. IgA class TGA were measured using either standard enzyme‐linked immunosorbent assay (Celikey, Phadia AB; cutoff > 7 U/L) or automatized EliA (Phadia; >7 U/L). The corresponding immunoglobulin G class antibody tests were used in case of IgA deficiency.

No‐biopsy criteria for CeD were introduced into Finland in 2018,^6^ and none of the study patients were diagnosed based solely on serology. All patients underwent gastroesophagoduodenoscopy with systematic sampling from the duodenum. The diagnosis was based on the demonstration of villous atrophy and crypt hyperplasia in well‐oriented biopsy specimens. Severity of mucosal damage was further categorized as partial, subtotal, and total atrophy; this classification has been used in clinical practice since the 1980s. These categories correspond to the Marsh‐Oberhuber grades IIIa, IIIb, and IIIc.

### 2.5 Adherence to GFD

Adherence to GFD at the most recent follow‐up visit was based on self‐report. Patients with strict GFD or only occasional (less than once a month) dietary lapses were defined as “adherent” and those with more frequent lapses as “nonadherent.” Results of CeD serology and duration of GFD were also recorded.

### 2.6 Statistical analysis

Quantitative data are reported as medians with quartiles and categorial variables as percentages. Cross‐tabulation with chi‐square (*χ* ^2^) or, when the assumptions for *χ* ^2^ were not met, with Fisher's exact test, was used to detect differences in categorial variables. Skewed variables were compared using the Mann–Whitney *U*‐test. *P*‐value of <0.05 was considered significant. All analyses were performed using SPSS, version 29 (IBM Corp.).

## 3 RESULTS

Altogether 125 (6.9%) CeD patients had a co‐existing T1D. One was excluded because of an unclear CeD diagnosis, 26 due to missing diagnostic data, 14 due to missing follow‐up data, and 15 because CeD had been diagnosed before T1D. Thirty‐one (45%) of the remaining patients were females. Median age at T1D diagnosis was 6.0 years (interquartile range: adherent 2.5–10.0, nonadherent 5.0–8.5) and at CeD diagnosis 8.8 years (5.0–12.2; 7.8–12.0). In 22 patients, CeD was diagnosed in connection with T1D based on routine testing, while in 47 patients CeD was diagnosed later (Table 1).

**Table 1:** Characteristics at CeD diagnosis in patients who have concomitant CeD and T1D and are adherent or nonadherent to GFD.

	Adherent, *n* = 50		Nonadherent, *n* = 19		
	*n*	%	*n*	%	*p*‐Value
Girls	22	44.0	9	47.4	1.000
T1D/CeD diagnosis concurrently	18	36.0	4	21.1	0.265
T1D diagnosed first	32	64.0	15	78.9	0.265
Family risk for CeD^1^	16	32.0	4	21.1	0.399
Family risk for T1D^1^	8	16.0	4	21.1	0.725
Other chronic disease^2^	18	36.0	10	52.6	0.275
	Median	Quartiles	Median	Quartiles	
Age at CeD diagnosis, years	8.0	5.0, 12.2	9.1	7.8, 12.0	0.413
Age at T1D diagnosis, years	4.8	2.5, 10.0	7.0	5.0, 8.5	0.454
Time between diagnoses, years	2.0	0.3, 4.8	2.5	0.8, 4.8	0.410
TGA at CeD diagnosis, U/mL	47.8	18.5, 120.0	120.0	24.0, 120.0	0.434
EmA at CeD diagnosis, titer	1:500	1:50, 1000	1:500	1:100, 4000	0.745
Follow‐up time on GFD, years	2.0	1.0, 6.0	2.0	1.0, 10.5	0.340
TGA at follow‐up visit, U/mL	3.3	1.5, 9.0	11.9	2.4, 20.8	0.107
EmA at follow‐up visit, titer	Neg	Neg, 1:5	1:5	Neg, 1:50	0.143

Abbreviations: CeD, celiac disease; EmA, serum endomysial antibodies; GFD, gluten‐free diet; T1D, type 1 diabetes; TGA, tissue transglutaminase antibodies.

^1^ First or second degree relative.

^2^ For example asthma, autoimmune thyroidal disease and Down syndrome.

**Figure 1:**
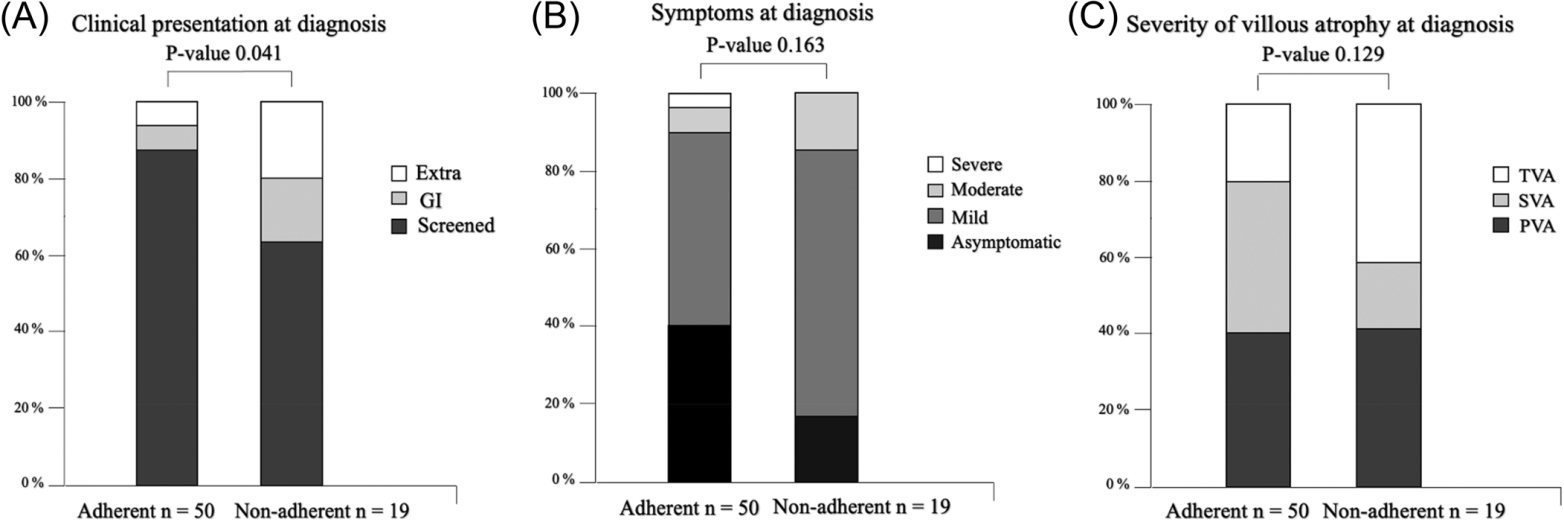
Clinical presentation (A), severity of symptoms (B), degree of small‐bowel mucosal villous atrophy (C) at CeD diagnosis in patients adherent and nonadherent to gluten‐free diet. Screen‐detected patients were found during annual routine antibody screenings independent of the presence or absence of possible symptoms. CeD, C eliac disease; Extra, extraintestinal symptoms; GI, gastrointestinal symptoms; PVA, partial villous atrophy; SVA, subtotal villus atrophy; TVA, total villous atrophy.

Median time on GFD was 2.0 (quartiles 1.0, 6.0) years. Altogether 50 children (72%) were categorized as adherent and 19 (28%) as nonadherent at the most recent visit. Adherent patients received their CeD diagnosis more often through preprogrammed screening, while the groups did not differ in severity of symptoms or duodenal damage at diagnosis (Figure 1). Overall, 81% of the patients were diagnosed through screening and 19% on the basis of symptoms. One screen‐detected patient also experienced possible CeD‐related symptoms but did not report them prior to testing, whereas those diagnosed outside the screening protocol reported symptoms that led to additional TGA testing.

The adherent and nonadherent patients likewise did not differs in sex, the chronological relation of the T1D and CeD diagnoses, family history of T1D or CeD, presence of other conditions, age at T1D or CeD diagnosis, time between the diagnoses, or EmA and TGA levels (Table 1). Furthermore, there was no significant difference between the groups in the duration of CeD follow‐up or antibody levels while on GFD (Table 1). At follow‐up, 30% of adherent and 32% of nonadherent patients with symptoms at diagnosis reported symptom relief on GFD.

All patients received dietary counseling at diagnosis and over 62% of the patients in both groups attended dietary counseling at least twice. The majority of the visits were individual appointments, and the rest were group appointments (data not shown).

## 4 DISCUSSION

Overall, 72% of CeD patients with concurrent T1D were adherent to strict GFD. This figure was somewhat lower than expected, as CeD is common and well‐known in Finland, GFD products are easily available, and children receive financial support to cover the additional costs. Adherence in particular was poorer than previously observed in Finnish screening‐detected patients, where strict adherence has been reported to be as high as 91%^9^ during long‐term follow‐up.^5^, ^10^ Compared to screening‐detected CeD patients in general, adherence in Finnish patients with a dual diagnosis have generally been slightly lower, varying 77%–86% in screening‐detected CeD patients with concomitant T1D.^5^, ^9^ Significantly more variable adherence rates, ranging from 33% to 92%, have been reported globally in patients with coexisting CeD and T1D.^7^, ^11^

Small cohorts and differing designs complicate comparisons but, according to our findings and previous by others, the burden of concurrent T1D may make GFD challenging to maintain.^7^, ^11^ Possible reasons for the increased burden may include, among others, reduced knowledge about CeD and the benefits of GFD, as well as experiences of restriction and social isolation.^7^, ^11^, ^12^ More frequent dietitian appointments may improve GFD adherence in individuals with a dual diagnosis, although this was not observed when gluten intake was objectively measured.^13^ Ultimately, the decision to participate in additional visits rests with the families. Identification of possible diet‐related psychological distress is important, and a multidisciplinary treatment approach may be beneficial.^12^ Of note, we used a strict definition of a GFD, considering even minor lapses, which may partly explain both the relatively high percentage of nonadherence and the lack of differences in serology. In other words, some individuals here classified as nonadherent may have only minimal deviations. However, a trend toward higher CeD antibody levels was observed in reportedly nonadherent patients. It must also be emphasized that serology may not detect occasional lapses, highlighting the need for better follow‐up tools.^14^ It is important to consider the limitations of the method used for assessing adherence; however, there is currently no clear evidence that any single approach is superior.^15^ Overall, our findings further confirm the need for special support for patients with a dual diagnosis of CeD and T1D.^1^

Then again, contrary to previous concerns that screen‐detected CeD patients exhibit poorer adherence to GFD,^16^ we found that adherent individuals were even more often identified through screening. In fact, the short term‐adherence of screening‐detected patients in Finnish cohorts has also previously^9^ been superior to that of patients detected on the basis of clinical suspicion, but in long‐term follow‐up, the former group has reported less strict adherence.^10^ This finding may indicate the need for support during the transition from pediatric to adult care. In any case, it is reassuring that screen‐detected diagnosis of CeD does not compromise treatment success even in T1D patients, especially given the growing interest in expanding screening for both conditions.^17^ The main reason that these patients are thought to be at risk for nonadherence is their presumed asymptomatic presentation, which could reduce motivation to maintain a restrictive GFD.^16^, ^18^ However, we observed no difference in this regard, and in fact, most individuals in both groups experienced symptoms. In some patients, symptoms may stem from other gastrointestinal diseases, nonceliac food intolerance, psychosomatic factors, or neurological disorders, with CeD being an incidental finding. In such cases, the lack of clear benefits of GFD may predispose to dietary lapses.^19^

Overall, the presentation of CeD may not be as important for dietary adherence as anticipated. It is also possible that screen‐detected patients only retrospectively attribute the symptoms to CeD, once they resolve on a GFD.^20^ Adherence may also be improved by the fact that the patients are informed about the possibility of a CeD at T1D diagnosis, allowing time for mental preparation. Even when CeD is screen‐detected concurrently with a T1D diagnosis, the initial focus is likely on the latter, giving patients time to adjust before starting GFD. On the other hand, after initial stabilization, in our clinical practice follow‑up for those with both T1D and CeD is usually incorporated into routine T1D visits. This integrated model may occasionally pose challenges, as the focus of follow‑up could shift more strongly toward T1D.

However, caution is needed when generalizing our findings, as maintaining a GFD may be more challenging outside Finland. CeD and T1D are also common here, which likely further facilitates adaptation to the diagnoses. In addition, in Finland, children with T1D and CeD receive a monthly financial allowance to help compensate for the additional costs of the disease. The possible effect of the cessation of this allowance during adolescence has not been studied. Adolescence is a risk period for nonadherence in CeD,^15^ including those with a dual diagnosis, although here age at diagnosis was similar between adherent and nonadherent participants. Although the groups did not differ in patient‐related factors, such as demographic data and familial risks for CeD and T1D, caution is warranted due to the relatively small sample size. For example, nonadherent patients showed a nonsignificant trend toward a higher prevalence of another chronic condition than did adherent patients. The presence of one more additional disease may further complicate adherence to GFD. Of note, excluding participants with inadequate follow‐up documentation may underestimate the severity of observed adherence issues. Additionally, self‐perceived reasons for nonadherence were not systematically collected, representing an important area for future studies. We have previously observed that, during long‑term follow‑up, the motivational factors behind adherence to a GFD appear to differ between individuals with and without concomitant T1D.^21^

In addition, given the constraints on healthcare resources, the transition from pediatric to adult care is an especially critical period, as substantial loss to follow‑up is increasingly documented during this time.^22^ Another important question is whether evaluation of adherence and quality of life using CeD‐specific tools should be integrated into clinical routine. Also, the optimal timing and frequency of visits remain to be determined and likely require individualization. For instance, adolescents may benefit from group appointments during a developmental stage in which peer support can be particularly valuable. Evaluating different follow‑up strategies and how they are implemented—both broadly and within specific clinical settings—is essential. More structured follow‑up models and focused research efforts are required to better support these patients and strengthen long‑term adherence.

## 5 CONCLUSION

To conclude, adherence to GFD is suboptimal in CeD patients with concomitant T1D, even in a high‐prevalence country with good availability of gluten‐free products. This finding further emphasizes the importance of special dietary support for children with a dual diagnosis of CeD and T1D. However, a screen‐detected CeD diagnosis does not appear to be harmful in this regard.

## CONFLICT OF INTEREST STATEMENT

Kalle Kurppa has received lecture fees from Thermo Fisher and Sanofi, and consulting fees from Takeda and Sanofi. Laura Kivelä has received a travel grant from Takeda. The remaining authors report no conflicts of interest.

## Supporting information

Supporting File 1.

## Data Availability

Research data may not be shared as they contain potentially identifying identifiable patient information.
