# Supplementary material for: Factors associated with adherence to gluten‐free diet in patients with concomitant celiac disease and type 1 diabetes
Source: JPGN Rep. 2026 Aug 14:10.1002/jpr3.70231. Online ahead of print. doi: 10.1002/jpr3.70231 (PMC13473925; doi:10.1002/jpr3.70231)
Supplement: Supplementary file 1 [file JPR3-9999-0-s001.docx]

**Supplementary figure 1.** Flow chart of the study population.

All patients in the original database

n = 1820

Excluded from the analysis:

- No T1D diagnosis, n=1694
- No biopsy proven CeD, n=1
- Age at T1D diagnosis not known, n=26
- Patients diagnosed with CeD first, n=15
- No long-term follow-up data in adherence available, n=14

n=69

Non-adherent

n=19

Adherent

n=50

**Supplementary Figure 1**. Flow chart of study population to demonstrate the exclusion criteria and formation of the final study population.
